# The Effect of Coaches’ Controlling Style on the Competitive Anxiety of Young Athletes

**DOI:** 10.3389/fpsyg.2017.00572

**Published:** 2017-04-12

**Authors:** Yago Ramis, Miquel Torregrosa, Carme Viladrich, Jaume Cruz

**Affiliations:** ^1^Research Group in Sport and Exercise Psychology, Department of Basic, Developmental and Educational Psychology, Universitat Autònoma de BarcelonaBarcelona, Spain; ^2^Research Group in Sport and Exercise Psychology, Department of Psychobiology and Methodology of Health Science, Universitat Autònoma de BarcelonaBarcelona, Spain

**Keywords:** sports, self-determined motivation, interpersonal style, structural equation modeling

## Abstract

Framed on a Self-Determination Theory perspective, the purpose of this study was to explore the predictive capacity of coaches’ interpersonal controlling style on the competitive anxiety of young athletes, considering the mediating effect of the athletes’ controlled motivation on this relationship. The sample consisted of 1166 athletes, aged between 9 and 18, who ranked their perceptions of coaches’ controlling style, as well as the reasons for participating in sport and their competitive anxiety before or during competition. The structural models assessing both the direct effect of the controlling style on the anxiety and the complete mediated effect of the controlled motivation on this relationship revealed good fit indices. However, a significant difference of the chi-square was obtained when comparing these models to the partial mediation model, providing evidence of this last model to be more adequate to describe the relationship between coaches’ controlling style and athletes’ competitive anxiety. Positive significant effects of coach controlling style on the three forms of competitive anxiety were found (β*_CS*-*SA_* = 0.21, *p* < 0.001; β*_CS*-*W_*= 0.14, *p* < 0.001; β*_CS*-*CD_*= 0.30, *p* < 0.001) indicating that coach controlling style could be an antecedent for athletes’ anxiety in a direct way. Although this style also predicts athletes’ motivation to participate, this indirect path seems to predict competitive anxiety in a less clear way. We discuss our results facing them up to Vallerand’s hierarchical model postulates, focusing on the relevant influence of coaches on the young athletes’ experience in the sport context.

## Introduction

Youth sport coaches can be determinant on the motivational and emotional experience of young athletes (Baker et al., 2000; Vazou et al., 2006; Balaguer et al., 2012). Trait competitive anxiety has been defined as a systematic tendency to react with overactivation before or during competitions (Martens, 1977). Although the research of anxiety in sport has typically focused on its influence on performance (Hanton et al., 2008), when studying this construct in recreational or educational sports, and specifically in youth populations, research has considered competitive anxiety as an indicator of ill-being (Martens, 1977; Smith et al., 2006). In order to explain the different facets of competitive anxiety, the Multidimensional Anxiety Theory (Martens et al., 1990) specifies that this anxiety could appear both in a somatic form, as physiological and muscular activation, and in a cognitive way in terms of mental concerns based on uncertainty regarding the competition and personal performance. According to this cognitive dimension of competitive anxiety, Smith et al. (2006), suggested two different characteristics to be considered: (a) worry, labeled as a negative concern associated to poor performance in competition, and (b) concentration disruption, referred to the difficulties of focusing on key aspects of the competitive task.

Self-Determination Theory (SDT; Deci and Ryan, 2010) proposes that ill-being, as well as well-being, is linked to motivational processes and modulated by social environment. This perspective considers human beings as active responsible of their own personal growth, integrity, and well-being, but also considering environment and social agents as potential facilitators or barriers to satisfy individuals’ psychological basic needs (i.e., autonomy, competence, and relatedness). Consequently, the social environment could partially determine the types of motivation (i.e., behavioral regulations) experienced by individuals when involved in different activities. According to SDT, behavioral regulations are distributed along a continuum that goes from more self-determined motivation to non-regulation. Intrinsic motivation is located on the more self-determined end, describing individuals who participate in an activity because of the satisfaction derived from the participation itself; extrinsic motivation, refers to a form of motivation by which individuals perform an activity because of consequent benefits of participation, thus motivated by external aspects; and amotivation refers to non-regulation of the activity which is performed without any conscious motivation, and represents the less self-determined form. Extrinsic motivation is subdivided along the continuum from more to less self-determination in these forms: (a) integrated regulation in which the activity is performed because it is considered as part of the individual’s self; (b) identified regulation motivated by expectations of obtaining valuable benefits because of participating; (c) introjected regulation motivated by the internalization of external elements such as feelings of guilt and shame; and (d) external regulation motivated by external antecedents or consequents.

Even though, both theoretical reviews and empirical studies have suggested a re-structuration of the motivational continuum grouping *autonomous motivation* and *controlled motivation* (Ryan and Connell, 1989; Vallerand, 2007; Lonsdale et al., 2009; Deci and Ryan, 2010). Autonomous motivation refers to the regulation of the behavior that is perceived to be caused by own interests and, according to literature, would have positive consequences at cognitive, affective, and behavioral levels (e.g., well-being, mental health, performance). On the other hand, controlled motivation describes the regulation of the behavior which is perceived to be externally driven in response to external pressures or demands and, as proved by previous studies, would have negative consequences (e.g., anxiety, depression, low performance; Pelletier et al., 2002; Appleton and Hill, 2012).

Framed on the SDT principles, Vallerand’s Hierarchical Model of Intrinsic and Extrinsic Motivation (HMIEM; Vallerand, 2007) illustrates the environmental influence of social agents on the motivation of individuals as well as on its affective consequents. This model provides a network of relationships of these variables at three different levels of generality: (a) situational, referred to motivation for a specific activity performed by the individual in a given situation; (b) contextual, referred to the individual’s motivation toward a specific environment (e.g., sport); and (c) global, referred to the general motivational orientation of an individual in the interaction among multiple contexts. This proposal suggests that, although some social agents can have an impact on global motivation (e.g., parents), the authority figure in a given context would be the most influential in that specific context. According to this, in the field of sport, coaches’ interpersonal style and motivational climate may determine children’s sporting experience, as well as their intention to keep involved in sports and develop an active lifestyle (Amorose and Anderson-Butcher, 2007).

Under the proposal of Deci and Ryan (1987), interpersonal style of significant others may adopt two forms: on the one hand, autonomy support refers to the disposition to support freedom, implication, and the individual’s autonomy to make decisions, by means of facilitation of relevant information and reduction of external pressure; on the other hand controlling style refers to a pressuring and authoritarian way of acting based on the use of contingent extrinsic rewards and punishments to regulate individuals’ behavior. Different studies both in the field of sport and physical education have suggested that perceptions of autonomy support facilitate athletes and students’ tendency to develop more autonomous and intrinsic motivation, thus obtaining more positive consequences on well-being and performance (e.g., Standage et al., 2006). On the other hand controlling style seems to be crucial on the thwarting of needs and, consequently, to predict athletes and students’ ill-being (Bartholomew et al., 2011). Previous research proves that ill-being consequents would be better predicted by the presence of a need-thwarting environment (i.e., coach controlling style) than by the absence of an autonomy-supportive environment (i.e., autonomy support; Bartholomew et al., 2011).

**FIGURE 1:**
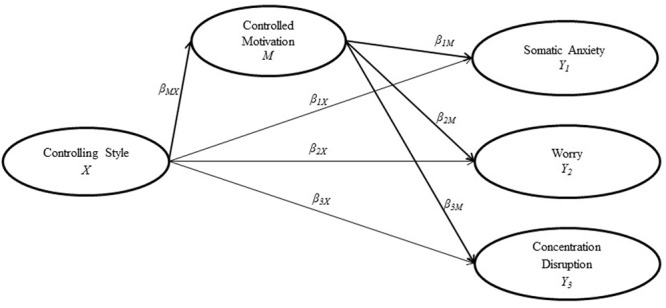
**Hypothesized structural model of Controlling Style, Controlled Motivation, and Competitive Anxiety**.

As competitive anxiety is a context specific distress that would systematically appear before or during competition, from a SDT perspective, it should be considered a contextual ill-being indicator. Taking into account the potential influence of coaches’ controlling interpersonal style, the purpose of this study was to evaluate the predictive capacity of coaches’ controlling style on athletes’ forms of somatic anxiety, worry, and concentration disruption. Additionally, we wanted to put in perspective the potential mediation of athlete’s controlled motivation on this relationship. Concerning the structural equation modeling approach to testing mediation, based on previous research on Vallerand’s HMIEM (Lonsdale et al., 2009) and taking in consideration that it is rather unlikely to find deterministic causal relations (i.e., complete mediation models) in general psychology, we hypothesized a partial mediation relation between the variables under study. In this regard, our hypothesis is that coach controlling style will positively predict the three forms of competitive anxiety and that this prediction will be significant both directly and mediated by the athletes’ controlled motivation. Additionally, we hypothesize that this coach controlling style will also positively predict controlled motivation and this motivation will positively predict athletes’ competitive anxiety (**Figure 1**).

## Materials and Methods

### Participants

Participants were 1166 Spanish athletes, whose ages ranged between 9 and 18 (*M* = 12.23; *SD* = 1.74). The sports included in the sample were football, basketball, handball, tennis, and synchronized swimming. A 15% of the participants were women. This distribution coincides with the general results of the “Study of Sporting Habits of the Scholar Population in Spain” (Consejo Superior de Deportes, 2011), concerning gender. All participants practiced regularly and competed in organized sports in their respective modality.

### Measures

#### Controlling Style

Coaches’ controlling interpersonal style was measured with the short seven item version of the *Controlling Coach Behaviors Scale* (CCBS; Bartholomew et al., 2010), adapted into Spanish by Castillo et al. (2014). This scale measures athletes’ perception of the controlling style of their coaches during practices and competitions. Participants rate a Likert scale from 1 (Completely False) to 5 (Completely True) sentences referred to their coaches’ usual behaviors (e.g., “My coach is less supportive of me when I’m not training and competing well”). High ratings on this questionnaire indicate that athletes perceive a high controlling style from their coaches, whereas low ratings imply low perceptions of this controlling style. Internal consistency for the present sample was tested both with Cronbach’s alpha (α = 0.67) and the inter-item mean correlation (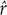 = 0.23).

#### Controlled Motivation

Athletes’ controlled motivation in the sport domain was assessed with the subscales of Introjected Regulation and External Regulation of the *Behavioral Regulation in Sports Questionnaire* (BRSQ; Lonsdale et al., 2008) adapted into Spanish by Viladrich et al. (2011). This instrument is used to measure the athletes’ motivation to participate in sport. Participants rate the eight items with the stem “I practice this sport...” (e.g., “because I would feel guilty if I quit”), using a 5-point Likert scale from 1 (Completely False) to 5 (Completely True). High ratings on this scale would indicate athlete’s higher controlled motivation to participate in sport, whereas low ratings would imply lower controlled motivation. Following previous literature in the use of this instrument to assess Controlled Motivation, the items of the Introjected and External Regulation were considered as a single scale referred as Controlled Motivation (Ntoumanis and Standage, 2009; Hodge and Lonsdale, 2011; Langan et al., 2015). Internal consistency of this scale was tested with Cronbach’s alpha (α = 0.81), and the inter-item mean correlation (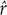 = 0.35).

#### Competitive Anxiety

Athlete’s competitive trait anxiety was evaluated with the Sport Anxiety Scale-2 (SAS-2; Smith et al., 2006) adapted into Spanish by Ramis et al. (2010). This scale is used to assess the athlete’s self-reported usual levels of Somatic Anxiety, Worry, and Concentration Disruption. Participants rate the 15 items with the stem “Before or while I compete in sports…” (e.g., my body feels tense), using a 4-point Likert scale from 1(Not at all) to 4(Very Much). High ratings on any of the subscales would indicate a high level of that anxiety form, whereas low ratings would imply lower anxiety symptoms. Internal consistency for the present sample was assessed with Cronbach’s alpha for the Somatic Anxiety (α = 0.81), Worry (α = 0.83), and the Concentration Disruption (α = 0.79), as well as with the inter-item mean correlation (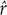 = 0.46; 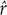 = 0.49; 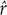 = 0.43, respectively) indicating a good reliability of the three subscales.

### Procedure

The current research was developed in accordance with the Ethical Principles of Psychologists and Code of Conduct of the American Psychological Association as well as the principles of the ethical board of our university. According to this Ethical Principles, informed consent may be dispensed where “research would not reasonably be assumed to create distress or harm (…) involving only anonymous questionnaires, (…) for which disclosure of responses would not place participants at risk of criminal or civil liability or damage their financial standing, employability or reputation, and confidentiality is protected” (American Psychological Association, 2002, p. 10). However, we considered adequate to obtain written informed consent from both participants and their parents or legal representatives in the case of underage youth.

#### Data Collection

Club coordinators and coaches were contacted and voluntarily accepted to participate in the study and days and times for the data collection were scheduled. Athletes attended to their clubs 20 min before their usual practice and answered the questionnaires in the changing room or in other adequate club facilities. In order to avoid any gender bias, questionnaires were grammatically adapted for girls and boys. Data collection protocol determined that at least two researchers should be present during the whole process to answer any eventual question. Athletes’ were previously informed about the aim of the study and the length of the data collection process, as well as about the confidentiality of the data. They all accepted voluntarily to participate in the research. Once they finished answering the questionnaires participants proceed with their usual practice routine.

#### Data Analytic Strategy

The data preparation, missing values analysis, data cleaning, and descriptive statistics were conducted using SPSS 17. Structural equation modeling was conducted using MPlus 7.4 to test both the measurement model of the different variables involved (i.e., estimate factor loadings allowing latent factors to freely correlate between them), and the hypothesis that controlled motivation would mediate the positive relationships between perceived coach controlling style and athletes’ competitive anxiety.

Due to the ordinal nature of the data and the presence of missingness, the weighted least squares mean and variance adjusted (WLSMV) estimator was used with pairwise deletion for missing values, both of them being the Mplus default for ordinal/categorical data. In concurrence with Graham (2009) and Muthén et al. (2011), the biases and loss of power attributable to this method can be considered inconsequential when the missingness is low. The goodness of fit indices were χ^2^, comparative fit index (CFI), Tucker–Lewis index (TLI), and root mean square error of approximation (RMSEA). Concerning quantitative indicators, CFI and TLI values > 0.95 and RMSEA < 0.06 are considered indicators of excellent fit (Hu and Bentler, 1999) and CFI and TLI values > 0.90 and RMSEA < 0.08 are considered acceptable (e.g., Marsh et al., 2013). Although the behavior of these cutoff values with categorical data remain under discussion (e.g., Yu, 2002) we employed these criteria in this study following previous studies in our field (e.g., Marsh et al., 2013).

As depicted in **Figure 1**, the partial mediation model posits that controlling style (*X*), would predict the controlled motivation of the athletes (*M*) by coefficient β*_MX_*, and this variable would predict as well the three forms of competitive anxiety (*Y*_*1*_, *Y*_*2*_, *Y*_*3*_) by coefficients β_*1M*_, β_*2M*_, β_*3M*_. A significant direct path between controlling style and competitive anxiety would also be expected by coefficients β_*1X*_, β_*2X*_, β_*3X*_, for partial mediation to be confirmed. In addition to the hypothesized model two alternative models were tested, the complete mediation model (in which β_*1X*_, β_*2X*_, and β_*3X*_ were deleted) and the direct effects model (in which β_*1M*_, β_*2M*_, and β_*3M*_ were deleted). As this models were more parsimonious and nested into the partial mediation model, the chi-square difference as computed in MPlus for categorical variables was conducted. However, as chi-square is sensitive to sample size, multiple authors have suggested that support for a more parsimonious model requires a change in CFI greater than 0.01 (Chen, 2007). Considering TLI and RMSEA, as they include a penalty for parsimony, the more parsimonious model can be accepted if it presents equal or better fit as the more restrictive model (Marsh et al., 2013). Variations in all four fit indices were considered.

## Results

### Preliminary Analyses

The initial screening of the data concerning missing values revealed no significant patterns of missing data in any of the subscales, being the loss of data less than 1%. In terms of item distributional assumptions the levels of Skewness (-3.58 to 1.70) and Kurtosis (-1.34 to 13.06) evidenced multivariate non-normality of data, thus giving support to the use of a robust estimator such as WLSMV.

### Descriptive Statistics

Descriptive statistics for perceived coach controlling style, controlled motivation, and competitive anxiety are presented in **Table 1**. All the mean scores were below the mid-point of the range for perceived Coach Controlling Style, Controlled Motivation, and Competitive Anxiety, with the exception of the SAS-2 Worry subscale.

**Table 1:** Descriptive statistics and reliability estimates.

	Rank	Mean (SD)	α	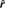
(1) Controlling Style	1–5	2.33 (0.76)	0.67	0.23
(2) Controlled Motivation	1–5	2.10 (0.92)	0.81	0.35
(3) Somatic Anxiety	1–4	1.94 (0.74)	0.81	0.46
(4) Worry	1–4	2.85 (0.82)	0.83	0.49
(5) Concentration Disruption	1–4	1.91 (0.69)	0.79	0.43

*SD, Standard Deviation; α, Cronbach’s alpha coefficient; 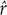, inter-item mean correlation.*

### Structural Equation Modeling

#### Measurement Model

The CFA was based on 30 indicators and 5 latent constructs. The fit indices for the measurement model, allowing the latent variables to freely correlate between them, provided an excellent fit to the data [χ^2^_(395)_ = 1192.34, *p* < 0.01; CFI = 0.96; TLI = 0.96; RMSEA = 0.04] according to Hu and Bentler’s (1999) cutoff criteria.

#### Test of Mediation

**FIGURE 2:**
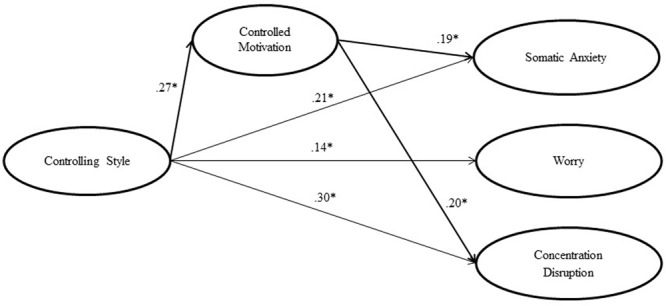
**Partial mediation model of Controlling Style, Controlled Motivation, and Competitive Anxiety.** ^∗^*p* < 0.001.

As exposed above, we tested the less parsimonious partial mediation model in first place, computing both the structural and the measurement model simultaneously. As showed in **Table 2**, the fit indices were good and both the direct path coefficients (β*_CS-SA_* = 0.21, *p* < 0.001; β*_CS-W_* = 0.14, *p* < 0.001, β*_CS-CD_* = 0.30; *p* < 0.001) and the mediated coefficients were significant (β*_CM-SA_* = 0.19, *p* < 0.001; β*_CM-CD_* = 0.20, *p* < 0.001), with the only exception of the Worry subscale, which was positively predicted by Coach Controlling Style but not by Controlled Motivation (β*_CM-W_* = 0.08, *p* = 0.028; see **Figure 2**). Successively, we tested the complete mediation model, by which the direct paths from Controlling Style to Competitive Anxiety were deleted. The comparison of this model with the partial mediation model revealed a significant chi-square difference and a ΔCFI over the cutoff point (-0.017) as considered by Chen (2007). Variations of TLI and RMSEA also gave support to the less parsimonious model. Finally, we tested the direct effects model by which the mediated paths were deleted. The fit indices were also excellent and, although the chi-square difference compared to the partial mediation model was significant, the variations of fit indices (ΔCFI = -0.008; ΔTLI = -0.009; ΔRMSEA = 0.004) were low. Considering that both partial mediation model and direct effects model showed excellent fit indices, we opted to select partial mediation model as the preferred model as it better fits the theoretical framework better. An additional analysis of the indirect effects of the partial mediation model was conducted in order to test the relative contribution of controlled motivation to the mediation of coach controlling style on competitive anxiety factors (**Table 3**) showing very low indirect effects of controlled motivation on somatic anxiety, worry, and concentration disruption.

**Table 2:** Fit statistics and standardized coefficient estimates for structural models.

Model	χ^*2*^	d.f.	Δχ^*2*^	Δd.f.	CFI	TLI	RMSEA	CS→Anx			CS→CM	CM→Anx		
								β_*1X*_	β_*2X*_	β_*3X*_	β_*MX*_	β_*1M*_	β_*2M*_	β_*3M*_
*Partial Medi*	1192.34	395	–	–	0.961	0.957	0.042	0.21^∗^	0.14^∗^	0.30^∗^	0.27^∗^	0.19^∗^	0.08	0.20^∗^
*Complete Medi*	1537.35	398	93.328^∗^	3	0.944	0.939	0.050	–	–	–	0.39^∗^	0.29^∗^	0.14^∗^	0.34^∗^
*Direc Eff*	1364.74	398	51.924^∗^	3	0.953	0.948	0.046	0.36^∗^	0.20^∗^	0.46^∗^	–	–	–	–

*Partial Medi, partial mediation model; *Complete Medi*, complete mediation model; *Direc Eff*, direct effects model; χ^*2*^= chi-square index; d.f., degrees of freedom; CFI, comparative fit index; TLI, Tucker–Lewis index; RMSEA, root mean squares error of approximation; CS, coach controlling style; CM, controlled motivation; Anx, competitive anxiety; β = path coefficients according to **Figure 1**; Som, Somatic Anxiety; Wor, Worry; CD = Concentration Disruption; ^∗^*p* < 0.001.*

**Table 3:** Standardized total and indirect effects for the partial mediation model.

	Total effects		Indirect effects	
	Estimate	95% CI	Estimate	95% CI
Controlling Style → Somatic Anxiety	0.266^∗^	0.224 to 0.529		
*Controlled motivation*			0.051^∗^	0.027 to 0.116
Controlling Style → Worry	0.166^∗^	0.120 to 0.453		
*Controlled motivation*			0.021	-0.007 to 0.078
Controlling Style → Concentration Disruption	0.359^∗^	0.356 to 0.713		
*Controlled motivation*			0.054^∗^	0.033 to 0.127

*CI, Confidence intervals; ^∗^*p* < 0.001. Italics refer to indirect effects of the model.*

## Discussion

The results of our study give support to the hypothesis that coaches’ controlling style positively predicts athletes’ perceived competitive anxiety under the mediation of controlled motivation. When considered as an exclusive predictor variable, this coach interpersonal style significantly predicted the occurrence of Somatic Anxiety, Worry, and Concentration Disruption. This result concurs with previous research in the field of coach interpersonal style on the perceived ill-being of athletes (Isoard et al., 2012). Furthermore, as we hypothesized, coach controlling style positively predicted athletes’ controlled motivation, however, when testing indirect effects of controlled motivation on the competitive anxiety of young athletes, results revealed that this indirect path does not have a determinant weight on this construct.

Despite this low indirect effects, the partial mediation model revealed the best fit indices giving support to the hypothesized model. These results suggest that, as raised by Bartholomew et al. (2011), coach controlling style is a good predictor of athletes’ controlled motivation and ill-being. Our results, based on a significant sample of young athletes, offer a general picture on how this relation may be. On the one hand, coaches’ controlling style, based on coercive, pressuring, and authoritarian acts (Deci and Ryan, 1987; Pelletier et al., 2002) could build a climate in which athletes feel the pressure to perform according to the coaches demands, leading to somatic symptoms and cognitive difficulties to focus on the competitive situation. On the other hand, this motivational climate based on the use of external rewards and controlling feedback could switch the goals of the athletes from more intrinsically to more externally regulated, and in the long term, lead to external burdens and anxious symptoms toward competition.

Analyzing the three factors of SAS-2 individually, significant direct effects of controlling style on somatic anxiety, worry, and concentration disruption were found. These results suggest that, when athletes feel pressured and thwarted by an authoritarian coach, they are most likely to display somatic signs of anxiety before and during competition. Additionally, as controlling coaches may deny attention and affection when desired behaviors are not displayed by their subordinates (Assor et al., 2004), athletes seem to be more concerned about poor performances and disappointing their coaches. Finally, negative or pressuring rapport developed by coaches seems to difficult athletes to focus on relevant cues of the game, as some authors have previously suggested (Baker et al., 2000).

Our study is framed on Vallerand’s HMIEM, which theoretically suggest a string of relationships between social agents, basic needs, motivation and motivational outcomes (Vallerand, 2007). Consequently, we aimed to evaluate not only direct effects of coach controlling style on competitive anxiety, but also if an indirect path, mediated by controlled motivation would better predict this relationship. Our results indicate that this mediated path also predicted somatic anxiety and concentration disruption, however, this mediated effects seem to be weak. Additionally, no significant mediated effect was found on the worry form of competitive anxiety.

The dissimilar functioning of the worry subscale in comparison with somatic anxiety and concentration disruption is not new. From a descriptive point of view, athletes have systematically reported higher scores on worry than on the other two subscales of the SAS-2, no matter the age of the participants (e.g., Grossbard et al., 2009). Besides, this factor has shown different correlation patterns with external variables (e.g., Grossbard et al., 2007). Previous studies have discussed on this differential psychometric functioning of the worry subscale, that might be describing a basal level of anxiety by which athletes report, not an anticipation of potential negative consequences, but a degree of appraisal of the personal importance and perceived responsibility regarding the task at hand (e.g., Lane et al., 1999; Ramis et al., 2015).

Although this research has confirmed significant predictions on competitive anxiety, as the design of the study is cross-sectional, the results should be taken with caution. In that sense, to assume any causal conclusions seems overstated. However, we believe that our study draws interesting interpretations on how social agents in sport might influence the emotional experience of young sport participants besides modeling their type of motivation. Further studies should complement these environmental antecedents with other dispositional variables related to the cognitive appraisal of the competition (Martinent and Ferrand, 2015), including for instance perfectionism (Appleton and Hill, 2012), or coping strategies (Dias et al., 2012), as well as to depict the antecedents of coaches’ motivation to develop one specific interpersonal style (Stebbings et al., 2011; Alcaraz et al., 2015a,b).

This study supports the relationship between coaches’ interpersonal style and competitive anxiety of young athletes under the mediation of athletes’ controlled motivation. The partial mediation model proved to have the best fit to the data when compared to the complete mediation model and the direct effects model. However, the forms of somatic anxiety and concentration disruption, but specially the worrying of young athletes, seem to be better predicted by the direct effects of coach controlling style than by the mediated path of controlled motivation, suggesting that more research is needed to understand the relationship between this factors and its motivational antecedents. Beyond this theoretical debate, practical implications of this study delve on the importance of educating youth sport coaches to develop more autonomy-supportive climates. Autonomy support, unlike controlling style, not only generates well-being outcomes on young athletes, but also prevents undesired outcomes such as forms of trait competitive anxiety.

## Author Contributions

YR has primarily conducted the research process, he’s been involved in the design of the study, data collection, data analysis and has guided the writing of the manuscript. MT has participated in the design of the research, defined the rationale of the study and collaborated in the consistency of the objectives along the manuscript. CV has selected and guided the methodology process and the adequate data analytic strategy. JC is responsible of the projects in which this study is integrated and he has collaborated in the definition of the rationale of the study. All authors have collaborated in the writing of the different sections and proofread its final version.

## Conflict of Interest Statement

The authors declare that the research was conducted in the absence of any commercial or financial relationships that could be construed as a potential conflict of interest.

## References

[B1] Alcaraz S. Torregrosa M. Viladrich C. (2015a). How coaches’ motivations mediate between basic psychological needs and well-being/ill-being. *Res. Q. Exerc. Sport* 86 292–302. 10.1080/02701367.2015.1049691 26230963

[B2] Alcaraz S. Viladrich C. Torregrosa M. Ramis Y. (2015b). Club and Players’ pressures on the motivation, vitality and stress of development coaches. *Int. J. Sports Sci. Coach.* 10 365–378. 10.1260/1747-9541.10.2-3.365

[B3] American Psychological Association (2002). *American Psychological Association Ethical Principles of Psychologists and Code of Conduct.* Available at: www.apa.org/ethics/code/principle

[B4] Amorose A. J. Anderson-Butcher D. (2007). Autonomy-supportive coaching and self-determined motivation in high school and college athletes: a test of self-determination theory. *Psychol. Sport Exerc.* 8 654–670. 10.1016/j.psychsport.2006.11.003

[B5] Appleton P. R. Hill A. P. (2012). Perfectionism and athlete burnout in junior elite athletes: the mediating role of motivation regulations. *J. Clin. Sport Psychol.* 6 129–145. 10.1080/10615800903330966

[B6] Assor A. Roth G. Deci E. L. (2004). The emotional costs of parents’ conditional regard: a self-determination theory analysis. *J. Pers.* 72 47–88. 10.1111/j.0022-3506.2004.00256.x 14686884

[B7] Baker J. Côté J. Hawes R. (2000). The relationship between coaching behaviours and sport anxiety in athletes. *J. Sci. Med. Sport* 3 110–119. 10.1016/S1440-2440(00)80073-0 11104303

[B8] Balaguer I. González L. Fabra P. Castillo I. Mercé J. Duda J. L. (2012). Coaches’ interpersonal style, basic psychological needs the well- and ill-being of young soccer players: a longitudinal analysis. *J. Sports Sci.* 30 1619–1629. 10.1080/02640414.2012.731517 23062028

[B9] Bartholomew K. J. Ntoumanis N. Ryan R. M. Bosch J. A. Thøgersen-Ntoumani C. (2011). Self-determination theory and diminished functioning: the role of interpersonal control and psychological need thwarting. *Pers. Soc. Psychol. Bull.* 37 1459–1473. 10.1177/0146167211413125 21700794

[B10] Bartholomew K. J. Ntoumanis N. Thøgersen-Ntoumani C. (2010). The controlling interpersonal style in a coaching context: development and initial validation of a psychometric scale. *J. Sport Exerc. Psychol.* 32 193–216. 10.1123/jsep.32.2.193 20479478

[B11] Castillo I. Tomás I. Ntoumanis N. Bartholomew K. J. Duda J. L. Balaguer I. (2014). Psychometric properties of the Spanish version of the Controlling Coach Behaviors Scale in the sport context. *Psicothema* 26 409–414. 10.7334/psicothema2014.76 25069563

[B12] Chen F. F. (2007). Sensitivity of goodness of fit indexes to lack of measurement invariance. *Struct. Equ. Model.* 14 464–504. 10.1080/10705510701301834

[B13] Consejo Superior de Deportes (2011). *Los Hábitos Deportivos de la Población Escolar en España.* Madrid: Consejo Superior de Deportes.

[B14] Deci E. L. Ryan R. M. (1987). The support of autonomy and the control of behavior. *J. Pers. Soc. Psychol.* 53 1024–1037. 10.1037/0022-3514.53.6.1024 3320334

[B15] Deci E. L. Ryan R. M. (2010). The “What” and “Why” of goal pursuits: human needs the self- determination of behavior the “What” and “Why” of goal pursuits: human needs the self-determination of behavior. *Psychol. Inq.* 11 227–268. 10.1207/S15327965PLI1104

[B16] Dias C. Cruz J. F. Fonseca A. M. (2012). The relationship between multidimensional competitive anxiety, cognitive threat appraisal, and coping strategies: a multi-sport study. *Int. J. Sport Exerc. Psychol.* 10 52–65. 10.1080/1612197X.2012.645131

[B17] Graham J. W. (2009). Missing data analysis: making it work in the real world. *Annu. Rev. Psychol.* 60 549–576. 10.1146/annurev.psych.58.110405.085530 18652544

[B18] Grossbard J. R. Cumming S. P. Standage M. Smith R. E. Smoll F. L. (2007). Social desirability and relations between goal orientations and competitive trait anxiety in young athletes. *Psychol. Sport Exerc.* 8 491–505. 10.1016/j.psychsport.2006.07.009

[B19] Grossbard J. R. Smith R. E. Smoll F. L. Cumming S. P. (2009). Competitive anxiety in young athletes: differentiating somatic anxiety, worry, and concentration disruption. *Anxiety Stress Coping* 22 153–166. 10.1080/10615800802020643 18937102

[B20] Hanton S. Neil R. Mellalieu S. D. (2008). Recent developments in competitive anxiety direction and competition stress research. *Int. Rev. Sport Exerc. Psychol.* 1 45–57. 10.1080/17509840701827445

[B21] Hodge K. Lonsdale C. (2011). Prosocial and antisocial behavior in sport: the role of coaching style, autonomous vs. controlled motivation, and moral disengagement. *J. Sport Exerc. Psychol.* 33 527–547. 10.1123/jsep.33.4.527 21808078

[B22] Hu L. Bentler P. M. (1999). Cutoff criteria for fit indexes in covariance structure analysis: Conventional criteria versus new alternatives. *Struct. Equ. Model.* 6 1–55. 10.1080/10705519909540118

[B23] Isoard S. Guillet E. Lemyre P.-N. (2012). A prospective study of the influence of perceived coaching style on burnout propensity in high level young athletes: using a self-determination theory perspective self-determination theory. *Sport Psychol.* 26 282–298. 10.1123/tsp.26.2.282

[B24] Lane A. M. Sewell D. F. Terry P. C. Bartram D. Nesti M. S. (1999). Confirmatory factor analysis of the competitive state anxiety inventory-2. *J. Sports Sci.* 17 505–512. 10.1080/026404199365812 10404499

[B25] Langan E. Hodge K. McGowan S. Carney S. Saunders V. Lonsdale C. (2015). The influence of controlled motivation alongside autonomous motivation: maladaptive, buffering, or additive effects? *Int. J. Sport Exerc. Psychol.* 14 1–15. 10.1080/1612197X.2015.1016084

[B26] Lonsdale C. Hodge K. Rose E. (2008). The behavioral regulation in sport questionnaire (BRSQ): instrument development and initial validity evidence. *J. Sport Exerc. Psychol.* 30 323–355. 10.1123/jsep.30.3.323 18648109

[B27] Lonsdale C. Hodge K. Rose E. (2009). Athlete burnout in elite sport: a self-determination perspective. *J. Sports Sci.* 27 785–795. 10.1080/02640410902929366 19437185

[B28] Marsh H. W. Nagengast B. Morin A. J. S. (2013). Measurement invariance of big-five factors over the life span: ESEM tests of gender, age, plasticity, maturity, and la dolce vita effects. *Dev. Psychol.* 49 1194–1218. 10.1037/a0026913 22250996

[B29] Martens R. (1977). *Sport Competition Anxiety Test.* Champaign, IL: Human Kinetics.

[B30] Martens R. Burton D. Vealey R. S. Bump L. A. Smith D. E. (1990). “Development and validation of the competitive state anxiety inventory-2” in *Competitive Anxiety in Sport* eds Martens R. Vealey R. S. Burton D. (Champaign, IL: Human Kinetics) 117–190.

[B31] Martinent G. Ferrand C. (2015). A field study of discrete emotions: athletes’ cognitive appraisals during competition. *Res. Q. Exerc. Sport* 86 51–62. 10.1080/02701367.2014.975176 25387279

[B32] Muthén B. O. Asparouhov T. Hunter A. M. Leuchter A. F. (2011). Growth modeling with nonignorable dropout: alternative analyses of the STAR^∗^D antidepressant trial. *Psychol. Methods* 16 17–33. 10.1037/a0022634 21381817 PMC3060937

[B33] Ntoumanis N. Standage M. (2009). Morality in sport: a self-determination theory perspective. *J. Appl. Sport Psychol.* 21 365–380. 10.1080/10413200903036040

[B34] Pelletier L. G. Fortier M. S. Vallerand R. J. Brière N. M. (2002). Associations among perceived autonomy support, forms of self-regulation, and persistence: a prospective study 1. *Motiv. Emot.* 25 279–307. 10.1023/A:1014805132406

[B35] Ramis Y. Torregrosa M. Viladrich C. Cruz J. (2010). Adaptación y validación de la versión española de la Escala de Ansiedad Competitiva SAS-2 para deportistas de iniciación. [Adaptation and validation of the Spanish version of the Sport Anxiety Scale SAS-2 for young athletes]. *Psicothema* 22 1004–1009. 21044545

[B36] Ramis Y. Viladrich C. Sousa C. Jannes C. (2015). Exploring the factorial structure of the sport anxiety scale-2: invariance across language, gender, age and type of sport. *Psicothema* 27 174–181. 10.7334/psicothema2014.263 25927698

[B37] Ryan R. M. Connell J. P. (1989). Perceived locus of causality and internalization: examining reasons for acting in two domains. *J. Pers. Soc. Psychol.* 57 749–761. 10.1037/0022-3514.57.5.749 2810024

[B38] Smith R. E. Smoll F. L. Cumming S. P. Grossbard J. R. (2006). Measurement of multidimensional sport performance anxiety in children and adults: the sport anxiety scale-2. *J. Sport Exerc. Psychol.* 28 479–501. 10.1123/jsep.28.4.479

[B39] Standage M. Duda J. L. Ntoumanis N. (2006). Students’ motivational processes and their relationship to teacher ratings in school physical education. *Res. Q. Exerc. Sport* 77 100–110. 10.1080/02701367.2006.10599336 16646357

[B40] Stebbings J. Taylor I. M. Spray C. M. (2011). Antecedents of perceived coach autonomy supportive and controlling behaviors: coach psychological need satisfaction and well-being. *J. Sport Exerc. Psychol.* 33 255–272. 10.1123/jsep.33.2.255 21558583

[B41] Vallerand R. J. (2007). “A hierarchical model of intrinsic and extrinsic motivation for sport and physical activity,” in *Intrinsic Motivation and Self-Determination in Exercise and Sport* eds Hagger M. S. Chatzisarantis N. L. D. (Champaign, IL: Human Kinetics) 255–363.

[B42] Vazou S. Ntoumanis N. Duda J. L. (2006). Predicting young athletes’ motivational indices as a function of their perceptions of the coach- and peer-created climate. *Psychol. Sport Exerc.* 7 215–233. 10.1016/j.psychsport.2005.08.007

[B43] Viladrich C. Torregrosa M. Cruz J. (2011). Calidad psicométrica de la adaptación española del cuestionario de regulación conductual en el deporte [Psychometric quality supporting the Spanish adaptation of the behavioral regulation in sport questionnaire]. *Psicothema* 23 786–794. 22047874

[B44] Yu C.-Y. (2002). *Evaluating Cutoff Criteria of Model fit Indices for Latent Variable Models with Binary and Continuous Outcomes.* Los Angeles, CA: University of California Los Angeles.

